# Visualization of the distal tibial plafond articular surface using four established approaches and the efficacy of instrumented distraction: a cadaveric study

**DOI:** 10.1007/s00068-022-01927-w

**Published:** 2022-03-17

**Authors:** Holger Kleinertz, Marlon Tessarzyk, Benjamin Schoof, Jakob Valentin Nüchtern, Klaus Püschel, Alexej Barg, Karl-Heinz Frosch

**Affiliations:** 1 Department of Trauma and Orthopaedic Surgery, University Medical Center Hamburg-Eppendorf, Hamburg, Germany; 2 Department of Trauma Surgery, Orthopaedics and Sports Orthopaedics, Asklepios Clinic St Georg, Hamburg, Germany; 3 Department of Legal Medicine, University Medical Center Hamburg-Eppendorf, Hamburg, Germany; 4 Department of Orthopaedics, University of Utah, Salt Lake City, Utah USA; 5 Department of Trauma Surgery, Orthopaedics, and Sports Traumatology, BG Hospital Hamburg, Hamburg, Germany

**Keywords:** Pilon fracture, Distal tibial plafond, Articular fracture, Surgical approach, Distraction, Visualization

## Abstract

**Purpose:**

Direct visualization is a very effective method in accomplishing adequate articular surface reconstruction in fracture repair. This study investigates distal tibial plafond articular surface visibility using the anteromedial, anterolateral, posteromedial, and posterolateral approaches, the effect of instrumented distraction on visibility, and which zones of the articular surface are visible for each approach.

**Methods:**

The anteromedial, anterolateral, posteromedial, and posterolateral approaches to the distal tibial plafond were performed on 16 cadaveric ankle specimens. The articular surface visualization for each approach was marked using an electrocautery device with manual and instrumented distraction. Articular surface visualization was photographically documented. Digital axial segmentation and quantitative analysis of the visualized distal tibial plafond articular surface were performed.

**Results:**

With manual distraction, distal tibial plafond articular surface visualization, expressed in percent of overall articular surface, was limited to 9% (SD ± 9) for the anteromedial, 24% (SD ± 18) for the anterolateral, 26% (SD ± 10) for the posteromedial, and 30% (SD ± 18) for the posterolateral approaches. Using instrumented distraction significantly improved articular surface visualization in all instances (*p* < 0.001). The anteromedial approach visible articular surface increased to 63% (SD ± 13), the anterolateral to 72% (SD ± 22), the posteromedial to 62% (SD ± 11), and the posterolateral to 50% (± 17).

**Conclusion:**

This study demonstrates the efficacy of instrumented distraction when attempting surgical visualization of the distal tibial plafond articular surface. Knowledge of approach specific articular surface visibility may assist the surgeon in choosing the appropriate approach(es) based on case-specific distal tibial plafond fracture patterns.

**Level of evidence:**

IV, cadaver study.

**Supplementary Information:**

The online version contains supplementary material available at 10.1007/s00068-022-01927-w.

## Introduction

Fractures of the distal tibial plafond, commonly referred to as pilon fractures, comprise approximately 5–10% of all tibial fractures. Complex pilon fractures are due to high-energy trauma, with axial compression forces causing disruption and comminution of the articular surface [1]. Open reduction and internal fixation (ORIF) is the widely accepted gold standard of pilon fracture treatment [2–5]. Despite advances in distal tibial plafond fracture treatment, like three-dimensional (3D) computed tomography (CT) for operative planning, intraoperative 3D imaging, and fixed angle implants, patients with complex fractures still have a high risk of poor outcomes [6–8]. Likewise, ankle injuries are the most common cause of ankle osteoarthritis (OA), with tibial plafond fractures contributing a remarkable amount considering their low incidence [9, 10]. Articular surface involvement has been shown to cut latency time between ankle injury and end-stage OA in half when compared to extraarticular fractures [11, 12].

Operative anatomic reconstruction and fixation of the articular surface is desired, as it is the most relevant factor in decreasing the incidence of ankle OA and poor outcomes [13–17]. Currently, most surgeons still rely on intraoperative 2D imaging assessing the anatomic reduction of a 3D structure. Thus, provided a satisfactory soft tissue status, the surgical approach(es) should allow for sufficient fracture exposure and articular surface visualization. Data related to distal tibial plafond visibility using open surgical approaches remains scarce and is limited to either expert experiences or data on the exposure of the posterior malleolus [18, 19]. To the best of our knowledge, there is no data that quantifies the efficacy of instrumented distraction in terms of improved articular surface visualization.

The purpose of this paper is to (1) assess distal tibial plafond articular surface visibility using four surgical approaches to the distal tibia, (2) evaluate the effect of instrumented distraction on articular surface visualization for all four approaches, and (3) give an approach-specific overview of the visibility of each of the nine defined zones.

## Materials and methods

This study was conducted with eight human whole-body cadaveric specimens, adding up to a total of 16 ankle specimens. Informed consent was obtained from a family member [20]. The procedures were performed within 48 h after death on non-frozen specimens. Inclusion of the cadaveric specimens required a BMI of ≥ 18 and ≤ 30 kg/m^2^, intact soft tissue around the ankle joint, no tibiotalar joint degeneration, and no signs of prior ankle injury or surgery. Ankle range of motion and stability also had to be within normal limits after loosening the rigor mortis.

The study was approved by the local ethics committee and conducted in accordance with the ethical standards laid out by the 1964 Declaration of Helsinki.

### Surgical approaches

All surgical approaches were performed by the same team of experienced and fellowship trained orthopaedic trauma surgery specialists.

The anteromedial (AM), anterolateral (AL), posteromedial (PM), and posterolateral (PL) approaches were performed on each specimen. To easily distinguish the markings and reduce the effects of soft tissue compromise, only two adjacent approaches were performed per ankle. Therefore, the AM approach was paired with the PL, and the AL approach was paired with the PM. Anterior approaches were performed in supine position and posterior approaches in prone position. To reduce errors caused by the sequence in which the approaches were performed, four procedures were first performed from anterior, and four procedures were first performed from posterior.

All approaches were conducted according to the principles laid out by the AO Foundation using the AO surgery reference [21–24]. The AM approach was carried out through an incision beginning 7 cm proximal to the ankle joint just lateral to the tibial crest and extending distally, following the medial border of the anterior tibial tendon ending at the anteromedial border of the navicular. Deep dissection was carried out just medial to the anterior tibial tendon and the joint capsule was opened in a sagittal direction [21]. For instrumented distraction, the Schanz screw was placed under direct visualization at the medial aspect of the talar body below the articular surface running from medial to lateral.

The AL approach was performed with a straight incision beginning 7 cm proximal to the ankle joint between the tibia and fibula and extending distally to the base of the fourth metatarsal. The superficial peroneal nerve was visualized and protected [25]. The fascia over the anterior compartment was incised and the joint capsule exposed by retracting the superficial peroneal nerve and anterior compartment tendons medially [18, 22]. Schanz screw placement for instrumented distraction was performed under direct visualization at the anterolateral aspect of the talar body below the articular surface running from lateral to medial.

For the PM approach, the 10 cm incision was centered between the posteromedial border of the distal tibia and the medial border of the Achilles tendon. The incision had a slight curve following the path of the posterior tibial tendon. Deep dissection visualized the muscle fibers of the flexor hallucis longus (FHL), which were retracted posterolaterally with the neurovascular bundle retracted anteromedially to expose the ankle joint [23]. The Schanz screw for instrumented distraction was placed under direct visualization at the medial aspect of the posterior talus below the articular surface running from medial to lateral.

The PL approach was performed through a 10 cm longitudinal incision halfway between the posterior border of the distal fibula and the lateral border of the Achilles tendon. Care was taken not to damage the sural nerve. The deep fascia was incised in line with the skin incision. The peroneal muscles were retracted laterally while the FHL was retracted medially [24, 26, 27]. For instrumented distraction, the Schanz screw was placed percutaneously at the calcaneus running from medial to lateral.

### Marking of the articular surface

For each approach, the visible articular surface using only manual traction combined with plantar or dorsiflexion was marked using an electrosurgical generator (Valleylab Force FX™, Medtronic, MN, USA) with a monopolar electrosurgical pencil (Erbe, Germany) and an angled micro-needle electrode (Valleylab™, Medtronic, MN, USA). To easily distinguish the anterior from the posterior visualized surfaces, dotted and solid lines were used, respectively.

Following manual traction, an external distractor (Large Distractor – Tibia, DePuy Synthes, IN, USA) was attached according to AO Foundation principles by inserting a 5 mm Schanz pin in the tibia and talus (see above) for the AL, AM, and PM approaches, as well as the calcaneus for the PL approach. Tibio-talar or tibio-calcanear distraction was applied and used to widen the joint space to 5 mm [28]. Again, the visible articular surface was marked using the monopolar electrocautery device.

After all four approaches were performed (two per ankle) and distraction was released, ankle stability was verified by manual examination before the ankle joints were disarticulated medially. The tibial plafond markings were documented via a digital photograph taken axially (ILCE-6500, SEL18135, Sony Corp., Tokyo, Japan) with a metric scale ruler set at the level of the articular surface for scale.

### Data analysis

**Fig. 1:**
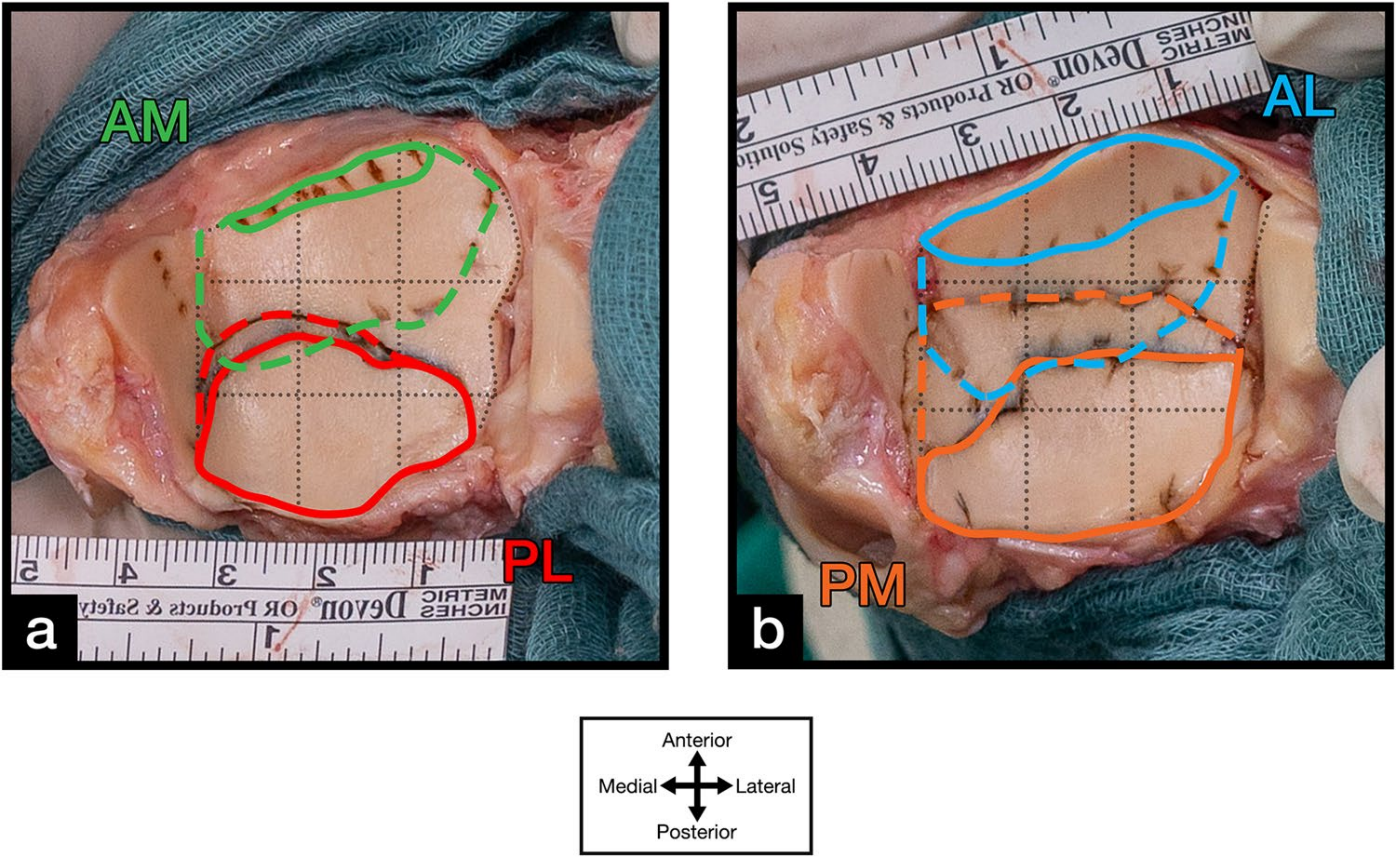
Exemplary specimens of the distal tibial plafond articular surface for the **a** anteromedial (AM) and posterolateral (PL) and the **b** anterolateral (AL) and posteromedial (PM) approaches with manual (solid line) and instrumented (dotted line) distraction

**Fig. 2:**
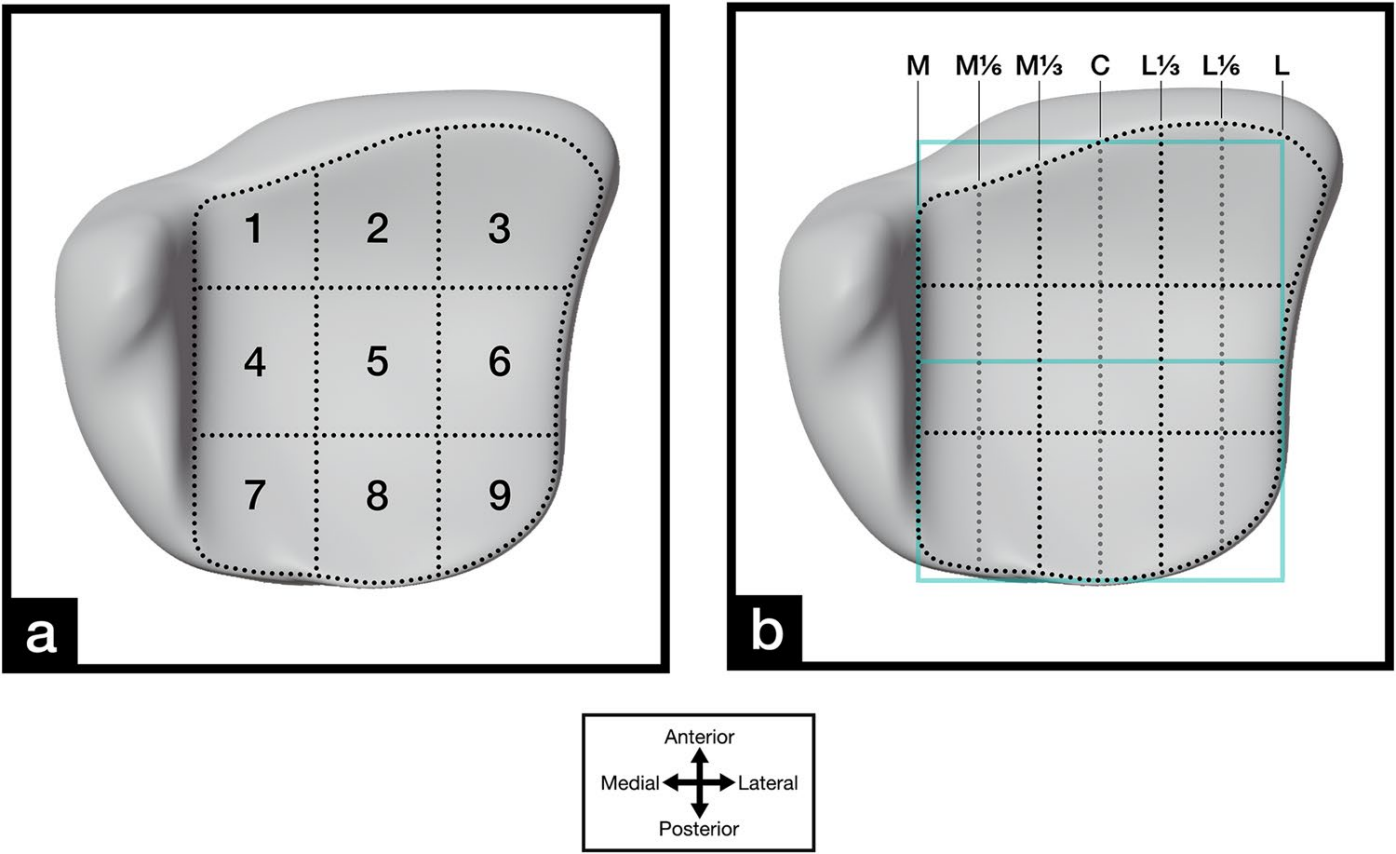
A standardized 3D model of the distal tibial plafond articular surface with **a** a standardized 3 × 3 grid and **b** seven defined anterior–posterior (AP) lines labeled from medial to lateral (medial (M), M1/6, M1/3, center (C), lateral (L)1/3, L1/6, and L). The lines were determined by dividing the AP center of the articular surface into 6 equally separated segments and used to measure the visible AP distance

Data analysis was performed digitally. The photos of the marked distal tibial plafond were imported into Adobe Photoshop (Photoshop Desktop version 21, Adobe, San Jose, CA, USA). The articular surface itself, excluding the medial malleolus, and the visible articular surface were digitally traced for each approach with manual and instrumented distraction (Fig. 1). Furthermore, a 3 × 3 grid was created to divide the distal tibial plafond into nine zones (1 = anteromedial, 2 = anterocentral, 3 = anterolateral, 4 = centromedial, 5 = central, 6 = centrolateral, 7 = posteromedial, 8 posterocentral, 9 = posterolateral) as previously described by other authors [29, 30] (Fig. 2). The grid was set to predefined landmarks to achieve reproducible results. The medial border was set parallel to the medial malleolus. The lateral border was set where the central horizontal line intersected with the lateral border of the articular surface. The anterior and posterior borders were set to where the central sagittal line intersected with the anterior und posterior borders of the articular surface (Fig. 2b).

Quantitative analysis was performed by analyzing the digitally traced images with ImageJ [31]. The scale was set according to the metric scale photographed at the level of the articular surface. The overall articular surface area, the marked articular surface area, the surface area of each of the nine zones, the marked articular surface area of each of the nine zones, as well as the visible distance for defined points on the anterior–posterior (AP) axis (medial (M), M1/6, M1/3, center (C), lateral (L)1/3, L1/6, and L) were assessed for each approach (Fig. 2b). Blender 3D modeling and rendering software (Blender version 2.81, Stitching Blender Foundation, Netherlands) was used to graphically illustrate the distal tibial plafond (Fig. 2).

### Statistical analysis

Statistical analysis was performed using GraphPad Prism 5.0 (GraphPad Software Inc., La Jolla, CA, USA). Normality testing of the visualized articular surface area and distance data sets was performed by utilizing the Kolmogorov–Smirnov test. The paired *t* test was used to determine significant differences in articular surface area and distance visibility with manual and instrumented distraction. Comparison of articular surface area visibility for each of the nine individual zones was performed using the Wilcoxon signed-rank test.

## Results

Overall, eight cadaveric specimens (7 males and 1 female), adding up to 16 ankle specimens, were included in the study. The average age was 61.1 years (range 48 – 77 , SD ±12.2) and average BMI was 25.1 kg/m^2^ (range 18.61 – 29.51 , SD ±3.7 ). The mean total surface area of the tibial plafond was 871 mm^2^ (range 587 – 1121, SD ±167). The AP distance for the tibial plafond articular surface averaged 23 mm (SD ± 3) for the M line, 30 mm (SD ± 3) for the M1/3 line, 31 mm (SD ± 4) for the L1/3 line, and 30 mm (SD ± 4) for the L line (Tables 1 and 2).

**Table 1:** Visualization of the distal tibial plafond articular surface and visible anterior–posterior distances using the anterior approaches with manual ( – ) vs. with instrumented ( +) distraction

		Total average	Anteromedial			Anterolateral		
			–	+	*p* value	–	+	*p* value
Surface	Area (mm^2^)	871 ± 167	69 ± 53	528 ± 147	.0001	195 ± 111	647 ± 225	.0009
	Percent (%)	–	9 ± 9	63 ± 13	< .0001	24 ± 18	72 ± 22	.0002
Distance	Medial							
	Distance (mm)	23 ± 3	1 ± 1	13 ± 2	< .0001	1 ± 1	15 ± 7	.0007
	Percent (%)	–	3 ± 7	57 ± 13	< .0001	2 ± 4	65 ± 34	.0012
	1/6 Medial							
	Distance (mm)	27 ± 3	3 ± 3	19 ± 3	< .0001	5 ± 6	19 ± 8	.0024
	Percent (%)	–	14 ± 15	73 ± 13	< .0001	19 ± 25	71 ± 32	.0027
	1/3 Medial							
	Distance (mm)	30 ± 3	5 ± 3	22 ± 4	< .0001	8 ± 6	22 ± 7	.0009
	Percent (%)	–	16 ± 12	75 ± 14	< .0001	27 ± 24	74 ± 21	.0006
	Center							
	Distance (mm)	31 ± 4	5 ± 5	22 ± 4	< .0001	9 ± 6	23 ± 5	.0004
	Percent (%)	–	17 ± 17	72 ± 12	< .0001	29 ± 23	74 ± 17	.0002
	1/3 Lateral							
	Distance (mm)	31 ± 4	3 ± 4	21 ± 4	< .0001	10 ± 6	24 ± 5	.0004
	Percent (%)	–	9 ± 16	68 ± 9	< .0001	32 ± 22	75 ± 15	.0002
	1/6 Lateral							
	Distance (mm)	30 ± 3	1 ± 2	16 ± 6	.0001	7 ± 4	23 ± 5	< .0001
	Percent (%)	–	2 ± 5	56 ± 19	< .0001	25 ± 14	74 ± 19	< .0001
	Lateral							
	Distance (mm)	30 ± 4	0 ± 0	6 ± 7	.0504	2 ± 3	14 ± 9	.0026
	Percent (%)	–	0 ± 1	22 ± 25	.0456	6 ± 9	48 ± 34	.0035

*P* values indicate statistically significant differences between both conditions. For the definition of distance, see Fig. 2b

**Table 2:** Visualization of the distal tibial plafond articular surface and visible anterior–posterior distances using the anterior approaches with manual ( – ) vs. with instrumented ( +) distraction

		Total Average	Posteromedial			Posterolateral		
			–	+	*p* value	–	+	*p* value
Surface	Area (mm^2^)		235 ± 107	546 ± 108	.0001	248 ± 130	408 ± 106	.0001
	Percent (%)	As Table 1	26 ± 10	62 ± 11	.0002	30 ± 18	50 ± 17	.0002
Distance	Medial							
	Distance (mm)	As Table 1	2 ± 3	17 ± 2	< .0001	3 ± 4	11 ± 4	< .0001
	Percent (%)		11 ± 13	76 ± 11	< .0001	14 ± 18	50 ± 19	< .0001
	1/6 Medial							
	Distance (mm)		8 ± 6	22 ± 3	.0008	11 ± 4	17 ± 3	< .0001
	Percent (%)		28 ± 21	80 ± 10	.0008	41 ± 16	67 ± 14	.0002
	1/3 Medial							
	Distance (mm)		12 ± 4	22 ± 3	< .0001	12 ± 5	17 ± 4	.0378
	Percent (%)		40 ± 10	74 ± 8	.0002	42 ± 19	60 ± 19	.0279
	Center							
	Distance (mm)		12 ± 4	22 ± 2	< .0001	12 ± 6	18 ± 4	.0009
	Percent (%)		38 ± 9	72 ± 6	.0001	40 ± 21	60 ± 17	.0013
	1/3 Lateral							
	Distance (mm)		10 ± 7	19 ± 8	.0026	8 ± 7	15 ± 5	.0024
	Percent (%)		29 ± 20	60 ± 26	.0044	29 ± 25	49 ± 20	.0023
	1/6 Lateral							
	Distance (mm)		5 ± 6	14 ± 9	.0221	6 ± 6	9 ± 6	.0198
	Percent (%)		16 ± 20	45 ± 32	.0209	20 ± 22	31 ± 25	.0211
	Lateral							
	Distance (mm)		3 ± 6	8 ± 10	.0816	4 ± 5	6 ± 6	.0342
	Percent (%)		9 ± 18	30 ± 35	.0864	15 ± 20	22 ± 25	.0385

*P* values indicate statistically significant differences between both conditions. For the definition of distance, see Fig. 2b

### Visibility of the articular surface using the anteromedial, anterolateral, posteromedial, and posterolateral distal tibial surgical approaches

For the AM approach, a mean articular surface area visualization of 69 mm^2^ (range 23–189, SD ± 53) was achieved with manual distraction. Those observations translated into an overall articular surface area visualization of 9% (range 3–31, SD ± 9). The AP visible articular surface distance averaged 1 mm (SD ± 1) or 3% (SD ± 7) on the M line, 5 mm (SD ± 3) or 16% (SD ± 12) on the M1/3 line, 3 mm (SD ± 4) or 9% (SD ± 16) on the L1/3 line, and 0 mm (SD ± 0) or 0% (SD ± 1) on the L line (Table 1).

For the AL approach, the mean articular surface area visualized with manual distraction was 195 mm^2^ (range 41 – 374, SD ± 111). This represented 24% (range 6–52, SD ± 18) of the overall articular surface area. The visible AP distance was 1 mm (SD ± 1) or 2% (SD ± 4) on the M line, 8 mm (SD ± 6) or 27% (SD ± 24) on the M1/3 line, 10 mm (SD ± 6) or 32% (SD ± 22) on the L1/3 line, and 2 mm (SD ± 3) or 6% (SD ± 9) on the L line (Table 1).

For the PM approach, a mean articular surface area of 235 mm^2^ (range 85–404, SD ± 107) was visible using manual traction, which translates to 26% (range 12–39, SD ± 10). The visible articular surface distance was 2 mm (SD ± 3) or 11% (SD ± 13) on the M line, 12 mm (SD ± 4) or 40% (SD ± 10) on the M1/3 line, 10 mm (SD ± 7) or 29% (SD ± 20) on the L1/3 line, and 3 mm (SD ± 6) or 9% (SD ± 18) on the L line (Table 2).

For the PL approach, the mean visible articular surface area using manual traction was 248 mm^2^ (range 49–377, SD ± 130) or 30% (range 7–62, SD ± 18). The visible articular surface distance was 3 mm (± 4) or 14% (SD ± 18) on the M line, 12 mm (SD ± 5) or 42% (SD ± 19) on the M1/3 line, 8 mm (SD ± 7) or 29% (SD ± 25) on the L1/3 line, and 4 mm (SD ± 5) or 15% (SD ± 20) on the L line (Table 2).

For more detailed information regarding the visible distal tibial plafond articular AP distances, please see Tables 1 and 2.

### The effect of instrumented distraction during the four distal tibial surgical approaches on distal tibial plafond articular surface visibility

The use of instrumented distraction increased the visible distal tibial plafond articular surface area and AP distances for all approaches.

For the AM approach, articular surface area visualization was extended to 528 mm^2^ (range 392–835, SD ± 147) or 63% (range 47–83, SD ± 13) (p = 0.0001). The AP visible articular surface distance increased to 13 mm (SD ± 2) or 57% (SD ± 13) on the M line, 22 mm (SD ± 4) or 75% (SD ± 14) on the M1/3 line, 21 mm (SD ± 4) or 68% (SD ± 9) on the L1/3 line, and 6 mm (SD ± 7) or 22% (SD ± 25) on the L line. The increases were significant for the M (*p* < 0.0001), M1/3 (*p* < 0.0001), and L1/3 lines (*p* < 0.0001), but not for L line (*p* = 0.0504) (Table 1).

Using instrumented distraction for the AL approach increased the visible articular surface to 647 mm^2^ (range 224–915, SD ± 225) or 72% (range 30–91, SD ± 22) (*p* = 0.0009). Visible articular surface distances increased to 15 mm (SD ± 7) or 65% (SD ± 34) on the M line, 22 mm (SD ± 7) or 74% (SD ± 21) on the M1/3 line, 24 mm (SD ± 5) or 75% (SD ± 15) on the L1/3 line, and 14 mm (SD ± 9) or 48% (SD ± 34) on the L line. The increases were significant for the M (*p* = 0.0007), M1/3 (p = 0.0009), L1/3 lines (*p* = 0.0004), and L lines (*p* = 0.0026) (Table 1).

For the PM approach, the visible articular surface area was extended to 546 mm^2^ (range 400–704, SD ± 108) or 62% (range 43–74, SD ± 11) (*p* = 0.0001). The visible articular surface distance increased to 17 mm (SD ± 2) or 76% (SD ± 11) on the M line, 22 mm (SD ± 3) or 74% (SD ± 8) on the M1/3 line, 19 mm (SD ± 8) or 60% (SD ± 26) on the L1/3 line, and 8 mm (SD ± 10) or 30% (SD ± 35) on the L line. The increases were significant for the M (*p* < 0.0001), M1/3 (*p* < 0.0001), and L1/3 lines (*p* = 0.0026), but not for the L line (*p* = 0.0816) (Table 2).

The use of instrumented distraction for the PL approach increased articular surface area visualization to 408 mm^2 ^(range 230–535, SD ± 106), or 50% (range 28–81, SD ± 17) (*p* = 0.0001). The use of an external distractor extended the visible articular surface distances to 11 mm (SD ± 4) or 50% (SD ± 19) on the M line, to 17 mm (SD ± 4) or 60% (SD ± 19) on the M1/3 line, to 15 (SD ± 5) or 49% (SD ± 20) on the L1/3 line, and 6 (SD ± 6) or 22% (SD ± 25) on the L line. The increases were significant for the M (*p* < 0.0001), M1/3 (*p* = 0.0378), L1/3 (*p* = 0.0024), and L lines (*p* = 0.0342) (Table 2).

**Fig. 3:**
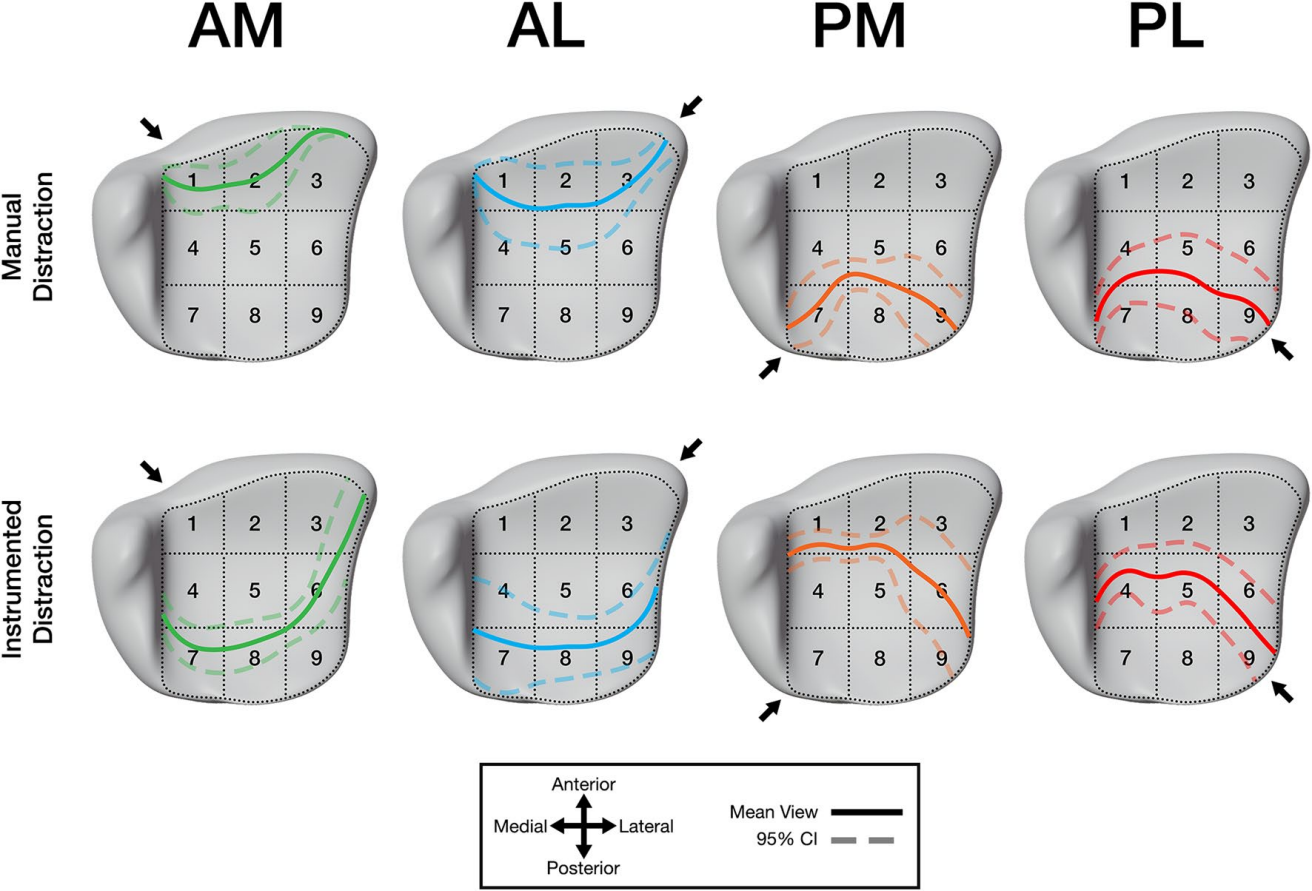
The mean visualization of the distal tibial plafond articular surface for the anteromedial (AM), anterolateral (AL), posteromedial (PM), and posterolateral (PL) approaches with manual and instrumented distraction. Solid lines represent the mean, dotted lines represent the 95% confidence interval (CI)

The mean visible articular surface distances (percent of total distance) in the AP projection and their 95% confidence intervals were transferred to our distal tibial plafond model. Figure 3 graphically exhibits the results of each approach with manual and instrumented distraction. For approach specific visible distances with manual and instrumented distraction for each individual patient, please see supplementary Fig. 1.

### A zone-specific overview of articular surface visibility using the four distal tibial surgical approaches

**Fig. 4:**
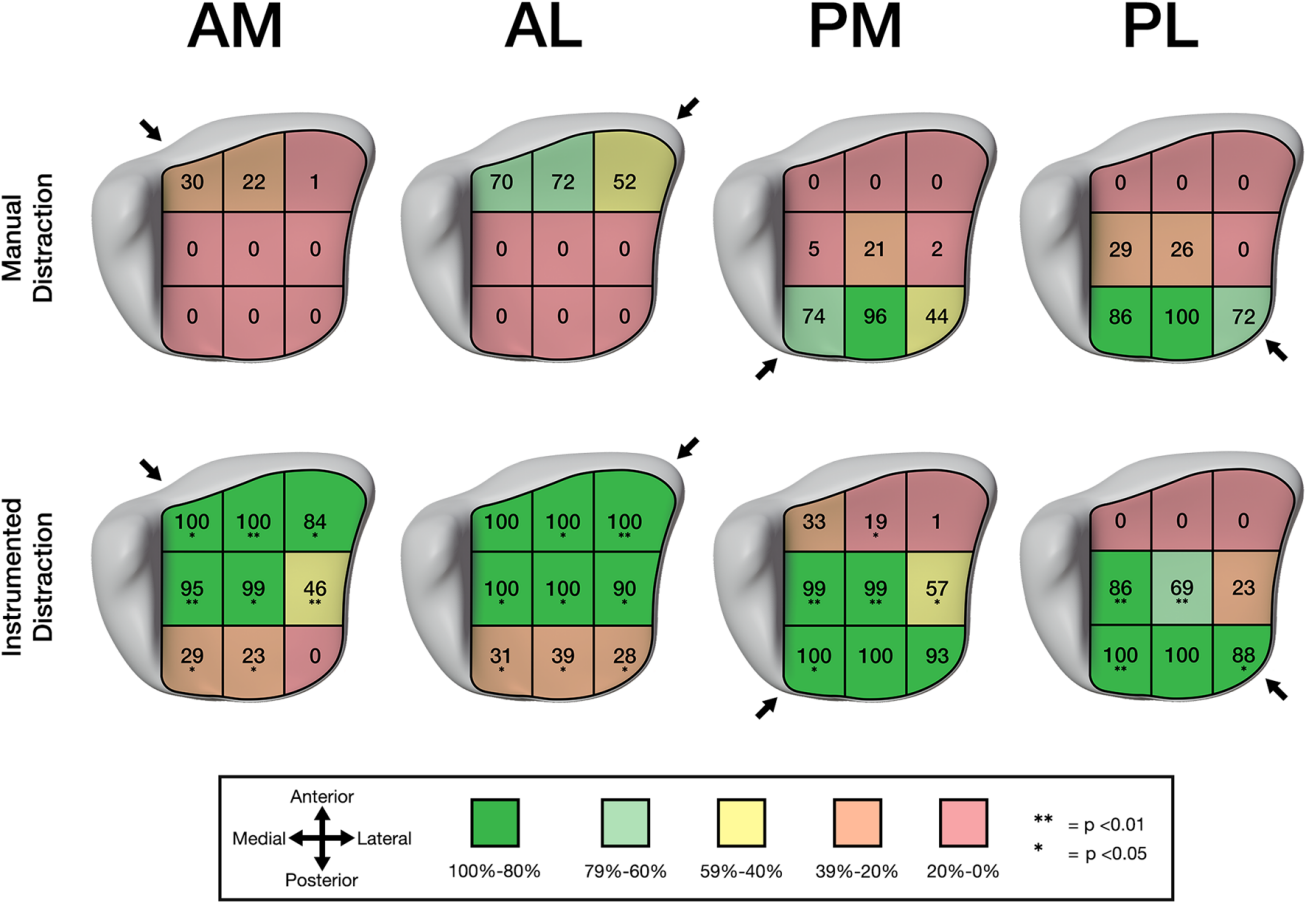
Visualization of the distal tibial plafond articular surface for each of the zones for the anteromedial (AM), anterolateral (AL), posteromedial (PM), and posterolateral (PL) approaches with manual and instrumented distraction. Values represent the median in percent. *P* values indicate statistically significant differences in visibility with manual and instrumented distraction

A 3 × 3 grid was created to divide the distal tibial plafond into nine individual zones. The median visible surface area of each of the nine zones was transferred to our distal tibial plafond model. Ranges of visibility were assigned a certain color to create a traffic light-like guide for the visibility of each zone using each approach (Fig. 4).

Visibility with the AM approach was very limited, with manual distraction showing only 30% and 22% of zones 1 and 2, respectively. External distractor usage significantly increased the visibility of each zone, except for zone 9. Overall, zones 1 through 5 showed good visibility using the AM approach combined with instrumented distraction.

For the AL approach, visibility was limited to the anterior three zones with manual distraction. Visibility of each zone significantly improved using instrumented distraction, except for zone 1. The AL approach allows good visualization of zones 1 through 6 using instrumented distraction, with zone 6 being the least exposed.

With manual distraction, the PM approach limits articular surface visualization to the three posterior zones, with only 44% of zone 9 being exposed. Using an external distractor significantly improved the visibility of zones 2, 4, 5, 6, and 7. Additionally, it allowed good exposure of posterior zones 4 through 9, with only limited exposure of zone 6.

Like the PM approach, the PL approach only allows for visualization of the posterior three zones, with posterolateral zone 9 showing the least exposure. Instrumented distraction significantly improved visualization of zones 4, 5, 7, and 9, allowing for good posterior exposure, but only limited visualization of centrolateral zone 6.

## Discussion

The present study demonstrates that visual exposure of the articular surface is very limited using only manual traction. This is especially true for the anterior approaches. The use of instrumented distraction increases articular surface visualization, particularly for the anterior approaches. Nevertheless, centrolateral zone 6 consistently remains difficult to expose.

The present study, for the first time, objectifies and quantifies articular surface visibility using the AM, AL, PM, and PL distal tibial plafond surgical approaches. Articular surface visibility with manual distraction is very limited utilizing the four approaches to access the distal tibial plafond. The AM and AL approaches allow for exposure of 9% and 24% of the articular surface, translating to limited exposure of zones 1 through 3. Overall exposure, even of anteromedial zone 1, is better with the AL approach. Zone 3 is not effectively visualized using the AM approach. The central and posterior zones 4 through 9 are not visible using either approach. Even though articular surface visualization is limited using the AM approach, its use has been suggested for fractures of the medial column of the distal tibia, as it enables access to the medial malleolus, the medial aspect of the anterior tibiotalar joint, and also allows for medial buttress plating [32]. Good visualization of the anteromedial zone 1 using the AL approach has already been described by other authors [18]. Therefore, in terms of articular surface visualization, the anterolateral approach should be the preferred choice when the medial and lateral anterior zones of the articular surface are involved.

The PM and PL approaches allow for exposure of 26% and 30% of the overall articular surface, respectively, with the exposure being limited to posterior zones 7 through 9. Posterolateral zone 9 visibility remains restricted using either approach. For a small series of type C pilon fractures with a displaced posterior malleolar fragment, Ketz et al. showed a lower rate of postoperative joint surface incongruency when using the PL approach in combination with an anterior approach, instead of an exclusively anterior approach with indirect reduction of the posterior malleolar fragment. The authors attribute this to better visualization of the posterior aspect of the distal tibia instead of direct articular surface visualization [27]. An accompanying lateral malleolar fracture can also be treated using the same skin incision as the PL approach [26, 27].

This study clearly demonstrates the positive effects of instrumented distraction on distal tibial plafond articular surface visibility for all approaches. Even though instrumented distraction is an invasive procedure, it is well established and its use in the surgical treatment of distal tibial plafond fractures has been suggested by various authors [2, 18, 27, 33]. In our study, the articular joint space was distracted to 5 mm without the use of extensive force to prevent neurovascular injuries, ligament elongation, and soft tissue irritation. Dowdy et al. showed that manual joint space distraction to an average of 4.2 mm (SD± 0.6) is safe to use. If any complications occurred, they were only associated with transient neurologic sensory changes [28]. We already used the 4 mm of distraction in a previous paper addressing the visibility of the posterior talar dome [34]. Other studies even suggest a distraction of up to 7–8 mm to be safe [35]. Nevertheless, a distraction of more than 5 mm did not necessarily lead to better visibility due to the curved anatomy of the ankle joint and increasing angulation of the bones when more distraction force was applied. Larger distraction may also result in neurovascular damage, which is often reversible, but still should be avoided. This is in line with our clinical experience where no soft tissue problems or neurovascular injuries could be observed up to now, indicating that a distraction distance of 5 mm is safe.

Especially for the anterior approaches, distractor placement is easy to perform. Pin placement is more complicated when the patient is in prone position. The smaller effect of instrumented distraction on the PL approach, when compared to the other three approaches, might be due to tibio-calcaneal pin placement, instead of tibio-talar pin placement for the AL, AM, and PM approaches. We decided for tibio-calcaneal pin placement in the PL approach since pin placement in the lateral talar neck in combination with medial tibial pin led to the femoral distractor crossing over the approach and obstructing the visibility and surgical intervention. We decided against closed talar pin placement due to the increased risk of neurovascular injury and to not compromise the vulnerable talar blood supply [36, 37]. Closed calcaneal pin placement is safe and allowed for medial application of the distractor where it would not interfere with the visibility and surgical intervention. Nevertheless, distraction to 5 mm was achieved in all cases without extraordinary distraction force.

Our observations on the effectiveness of instrumented distraction go along with those of other researchers [2, 18, 33]. Mehta et al. state that distractor placement, pharmacologic relaxation, headlamp use, and intraoperative imaging allow for visualization of the entire articular surface using the AL approach [18]. Even though we could not visualize the entire articular surface in any case (Supplementary Fig. 1), we had direct articular surface visualization of an average of 72% (SD ± 22) with a maximum articular surface visualization of 91% in patient one using the AL approach. This represented the best visibility of all four approaches indicating higher joint laxity laterally. The inferior visualization compared to the study by Mehta et al. may be explained by the difference in distraction force.

For the anterior approaches, visualization of zones 1 through 6 is significantly improved by instrumented distraction. For the posterior approaches, visualization of zones 4 through 9, except zone 8, is also improved. Therefore, instrumented distraction improves visualization of both the anterior and posterior zones and allows visual access to the central zones of the distal tibial plafond articular surface. Centrolateral zone 6 remains especially difficult to access with the anterior and posterior approaches, with the AL approach allowing the best visualization. Surgeons must be well aware of this fact since centrolateral zone 6 is commonly involved in fractures of the distal tibial plafond [38].

Though all experiments were carefully designed and meticulously analyzed, this study has certain limitations. Each approach was only performed a total of eight times due to the difficult nature of attaining cadaveric specimens that fulfilled our study requirements. Therefore, due to a limited number of specimens in each approach group, the results may be underpowered, which should be considered when interpreting them. One cadaver was 161 cm tall, appreciably  smaller than the average height of 180 cm. This specimen had the smallest articular surface with an area of 587 mm^2^. The AP distances were not as affected, as the articular surface was rather long and narrow. The central AP distance of this specimen was found to be 26 mm, compared to the average of 31 mm. However, we found the results to be in line with those of the other cadaveric specimens. Further, our study was performed on cadaveric specimens without muscular tone after the rigor mortis was cleared. This should closely resemble the findings in a patient in a surgical setting under anesthesia. Nevertheless, the real accessibility of the tibial plafond may be lower than observed in our study. Possible alterations in soft tissue properties caused by freezing and unfreezing were further minimized by using non-frozen cadaveric specimens no older than 48 h after death. Additionally, whole-body cadaveric specimens were used, which best resemble physiologic anatomic and biomechanical properties. In terms of analysis, articular surface exposure was assessed using two-dimensional (2D) digital photographs. Even though these were taken strictly perpendicular to the articular surface with a metric scale set at the level of the articular surface, 2D analysis of a 3D surface lacks accuracy in terms of exact dimensions. However, for the purpose of objectifying articular surface visualization, we found this approach applicable. Distal tibial plafond articular surface visualization could have most likely been improved by performing established osteotomies. As those procedures are invasive, associated with further complications, and not always feasible in a fractured ankle, they were not included in this study [39–41]. In contrast to a trauma surgery setting, the procedures were performed on uninjured ankles without soft tissue lacerations or swelling, which usually complicates and may even dictate the choice of approach in operative treatment [5, 42, 43]. Furthermore, this study does not consider the fracture morphology, and the necessity of a biomechanically favorable plate fixation. Surgeons must keep this in mind when planning their approach(es).

## Conclusion

Instrumented distraction improves distal tibial plafond articular surface visibility, which is very limited using only manual traction. Since the advantageous effects were more pronounced for the anterior approaches, our data suggest that when both an anterior and posterior approach are planned, instrumented distraction is more beneficial for the anterior approach. Overall, when using instrumented distraction, the AL and PM approaches allow for better articular surface visualization than the AM and PL approaches. Surgeons must be aware that centrolateral zone 6 is usually difficult to visualize, but is commonly involved in fractures.

## Supplementary Information

Below is the link to the electronic supplementary material.

Supplementary file1 (PDF 4326 KB) Fig. 1 Each specimen transferred to the standardized model of the distal tibial plafond for the anteromedial (AM), anterolateral (AL), posteromedial (PM), and posterolateral (PL) approaches with manual (solid line) and instrumented (dotted line) distraction
