# Supplementary material for: Visualization of the distal tibial plafond articular surface using four established approaches and the efficacy of instrumented distraction: a cadaveric study
Source: Eur J Trauma Emerg Surg. 2022 Mar 17;48(5):4031–41. doi: 10.1007/s00068-022-01927-w (PMC9532318; doi:10.1007/s00068-022-01927-w)
Supplement: Supplementary file 1 — Supplementary file1 (PDF 4326 KB) Fig. 1 Each specimen transferred to the standardized model of the distal tibial plafond for the anteromedial (AM), anterolateral (AL), posteromedial (PM), and posterolateral (PL) approaches with manual (solid line) and instrumented (dotted line) distraction [file 68_2022_1927_MOESM1_ESM.pdf]

Supplementary Figure 1

European Journal of Trauma and Emergency Surgery

Visualization of the distal tibial plafond articular surface using four established approaches and the efficacy of instrumented distraction: a cadaveric study

Holger Kleinertz[1], Marlon Tessarzyk[1], Benjamin Schoof[1], Jakob Valentin Nüchtern[2], Klaus Püschel[3], Alexej Barg[1,4,5], Karl-Heinz Frosch[1,5]

[1] Department of Trauma and Orthopaedic Surgery, University Medical Center Hamburg-Eppendorf, Hamburg, Germany

[2] Department of Trauma Surgery, Orthopaedics, and Sports Orthopaedics, Asklepios Clinic St Georg, Hamburg, Germany

[3] Department of Legal Medicine, University Medical Center Hamburg-Eppendorf, Hamburg, Germany

[4] Department of Orthopaedics, University of Utah, Salt Lake City, Utah, USA

[5] Department of Trauma Surgery, Orthopaedics, and Sports Traumatology, BG Hospital Hamburg, Hamburg, Germany

Corresponding Authors:

Holger Kleinertz
Department of Trauma and Orthopaedic Surgery, University Medical Center Hamburg-Eppendorf, Martinistr. 52, 20246 Hamburg, Germany.
h.kleinertz@uke.de, Tel: +49 (0) 40 7410 – 0

Alexej Barg
Department of Trauma and Orthopaedic Surgery, University Medical Center Hamburg-Eppendorf, Martinistr. 52, 20246 Hamburg, Germany.
al.barg@uke.de, Tel: +49 (0) 40 7410 – 0

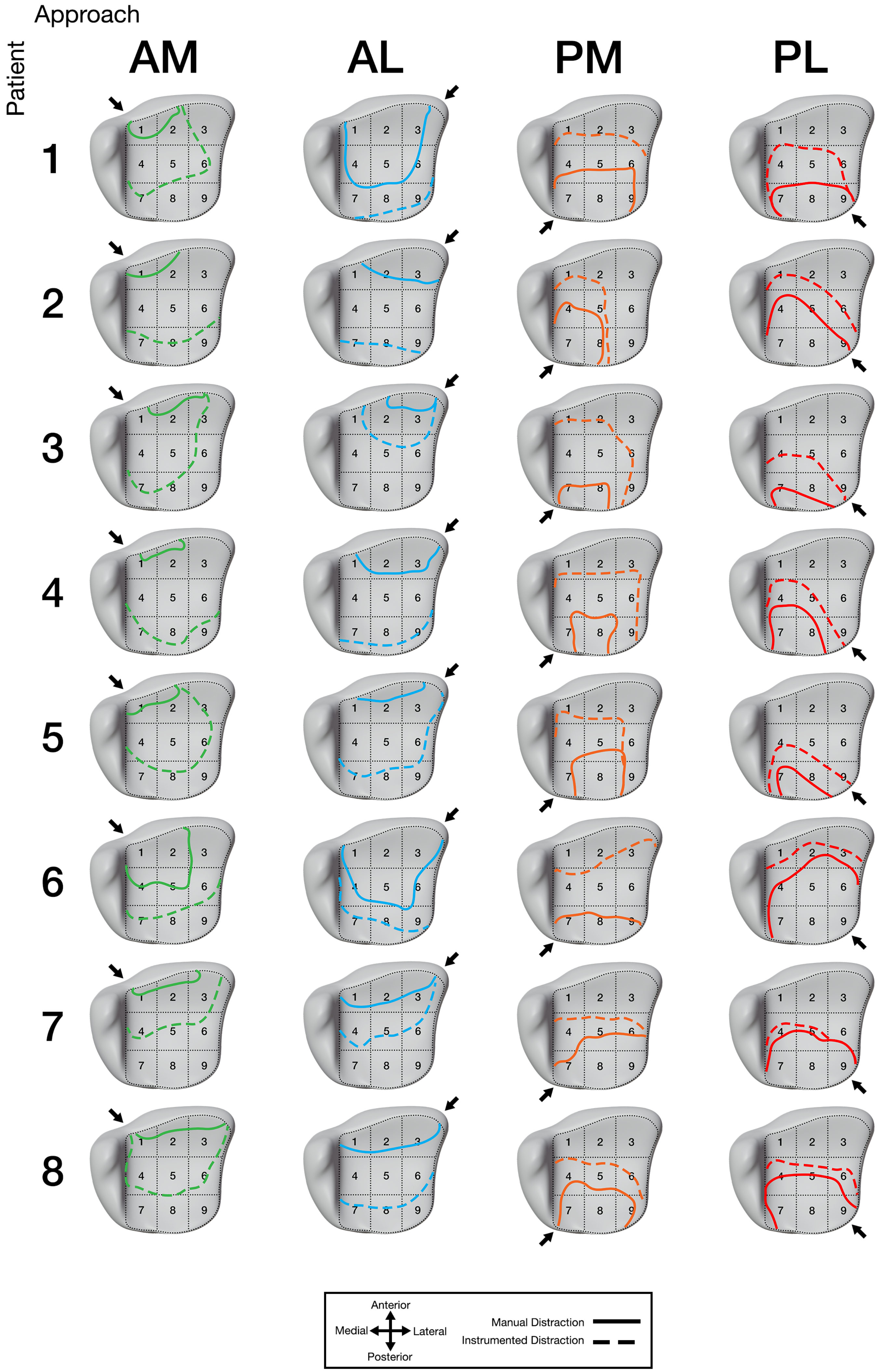
Approach
Patient
AM
AL
PM
PL
1
2
3
4
5
6
7
8
Anterior
Medial
Lateral
Posterior
Manual Distraction
Instrumented Distraction
